# Effects of Chemically Treated Eucalyptus Fibers on Mechanical, Thermal and Insulating Properties of Polyurethane Composite Foams

**DOI:** 10.3390/ma13071781

**Published:** 2020-04-10

**Authors:** Sylwia Członka, Anna Strąkowska, Piotr Pospiech, Krzysztof Strzelec

**Affiliations:** 1 Institute of Polymer and Dye Technology, Faculty of Chemistry, Lodz University of Technology, Stefanowskiego 12/16, 90-924 Lodz, Poland; anna.strakowska@p.lodz.pl (A.S.); krzysztof.strzelec@p.lodz.pl (K.S.); 2 Centre of Papermaking and Printing, Lodz University of Technology, Wolczanska 223, 90-924 Lodz, Poland; piotr.pospiech@p.lodz.pl

**Keywords:** polyurethanes, eucalyptus fibers, chemical treatment, insulating properties, mechanical properties

## Abstract

In this work, rigid polyurethane (PUR) foams were prepared by incorporating 2 wt% of eucalyptus fibers. The eucalyptus fibers were surface-modified by maleic anhydride, alkali, and silane (triphenylsilanol) treatment. The impact of the modified eucalyptus fibers on the mechanical, thermal, and fire performances of polyurethane foams was analyzed. It was observed that the addition of eucalyptus fibers showed improved mechanical and thermal properties and the best properties were shown by silane-treated fibers with a compressive strength of 312 kPa and a flexural strength of 432 kPa. Moreover, the thermal stability values showed the lowest decline for polyurethane foams modified with the silane-treated fibers, due to the better thermal stability of such modified fibers. Furthermore, the flame resistance of polyurethane foams modified with the silane-treated fibers was also the best among the studied composites. A cone calorimetry test showed a decrease in the peak of heat release from 245 to 110 kW∙m^−2^ by the incorporation of silane-treated fibers. Furthermore, total heat release and total smoke release were also found to decrease remarkably upon the incorporation of silane-treated fibers. The value of limiting oxygen index was increased from 20.2% to 22.1%. Char residue was also found to be increased from 24.4% to 28.3%. It can be concluded that the application of chemically modified eucalyptus fibers has great potential as an additive to incorporate good mechanical, thermal, and fire properties in rigid polyurethane foams.

## 1. Introduction

Polyurethanes (PURs) are one of the most versatile materials. Polyurethane foams are applied across several areas such as building, industrial insulation, construction, automotive, and domestic appliances [1] due to its excellent properties such as low thermal conductivity, good mechanical properties, and water resistance. 

As a structural material, rigid polyurethane foams should possess a certain strength, toughness, and heat resistance. Due to this, many additives such as nanoparticles or fibers have been used as reinforcement fillers for the production of rigid polyurethane foams [2,3,4,5,6,7,8]. Besides several inorganic fillers, such as nanoclay [9,10], expandable graphite [11,12,13,14], silica [15,16,17,18,19], talc [17,20,21], or polyhedral oligosilsesquioxanes (POSS) [22,23,24,25], the production of composite foams with bio-based fillers from natural recourses has attracted attention due to their environmentally friendly character [26,27,28,29]. 

Among different bio-based fillers, the chemical composition of eucalyptus fibers has great potential as a sustainable reinforcement for novel polyurethane composite foams. The chemical composition of eucalyptus involves cellulose (~48%), hemicellulose (~22%), and lignin (~22%) [30]. Eucalyptus is also a rich source of bioactive substances and metabolites (e.g., phenolic compounds and triterpenic acids). Due to their good quality, renewability, biodegradability, relatively low price and relevant mechanical properties, eucalyptus fibers have been used as a reinforcement material to produce polymeric composites [31,32,33]. It has been well documented that eucalyptus fibers can be employed as reinforcing fillers in polymer composites such as polyethylene, polystyrene, poly(vinyl chloride), and polypropylene. For example, Kumar et al. [34] showed that eucalyptus fiber reinforced epoxy composites exhibit higher flexural, tensile, and compression strength with increases in eucalyptus fiber content. Eucalyptus fibers were used as a reinforcing filler in epoxy and polyester matrices by [35]. The results showed that the incorporation of natural fibers slightly increased the elastic modulus in both matrices and slightly decreased the tensile strength in epoxy and polyester composites. Silva et al. [36] showed that the incorporation of eucalyptus sawdust filler results in improved mechanical and thermal properties, as well as limited water uptake. Polyamide-6 composites reinforced with semi-bleached eucalyptus fibers were also prepared by Fernandes et al. [37] Composites reinforced with 30 wt% of fibers exhibited minor improvements in the thermal and mechanical properties. 

Lignocellulosic fillers, including eucalyptus fibers, have been successfully used as reinforcing materials in polymeric matrices [38,39,40,41,42,43,44,45,46,47,48], however, the hydrophilic character of natural, organic fillers reduces their applicability [49]. The differences in chemical structures between hydrophilic fillers and hydrophobic matrices can result in poor adhesion, interphase separation, and the limited stress transfer of the composites [50]. Moreover, waxes and pectins that cover the surface of the fillers may act as a barrier and disrupt the effective interfacial adhesion between the functional groups of the filler and the matrix surface. Therefore, the chemical modification of organic fillers is a necessary step in order to prepare composites reinforced with natural fillers. Various investigations were carried out on the modifications of natural fillers to improve compatibility with the polymeric matrix [51,52,53]. Several authors reported different surface modifications for lignocellulosic fillers, which involve chemical modifications, such as acetylation [54], alkalization [55], benzoylation [56], grafting [56], and silane treatment [57]. For example, wood fibers were treated by a coupling agent (KH-550) by Du [58]. The author showed that chemically-treated fibers could significantly improve the interphase adhesion between polyimide matrix and wood fibers. Silane-treated fibers improved the worn and tensile properties of composites. Epoxy composites reinforced with alkali-treated (6% solution of NaOH) white/brown coir fibers were investigated by Valášek et al. [59]. The chemical treatment of fibers improved the interaction with the epoxy matrix and improved the mechanical properties of composites. Similar results were reported by Shalwan and Yousif [60], who synthesized epoxy composites reinforced with sodium hydroxide-treated date palm fibers. Such prepared composited were characterized by improved wear characteristics and mechanical properties. Chemical modifications, such as acetylation, mercerization, latex coating, peroxide treatment, permanganate treatment, and acrylonitrile grafting of oil palm fiber were investigated by Sreekala et al. [32]. The results showed that phenol formaldehyde composites reinforced with chemically-treated fibers exhibited improved impact resistance and better flexural strength. 

Many previous studies investigated the impact of chemically modified fillers on the mechanical and thermal properties of composites, however, no studies have been devoted to the investigation of the polyurethane foams reinforced with chemically-treated eucalyptus fibers. Because of this, the impact of chemically-treated fibers on morphological, mechanical, and thermal properties of polyurethane composites should be clearly defined. 

## 2. Experimental

### 2.1. Materials

Aromatic polyester polyol (STEPANPOL PS-2352, a hydroxyl value of 240 mgKOH/g, the functionality of 2) was purchased from Stepan Company. Polymeric diphenylmethane-4,4’-diisocyanate (PUROCYN B, 31% of isocyanate groups) was purchased from Purinova Sp. z o.o. Kosmos 75 (standard potassium octoate catalyst) and Kosmos 33 (standard potassium acetate catalyst) produced by Evonik Industries AG were used as catalysts. Silicone surfactant (TEGOSTAB B8513) was bought from Evonik Industries AG. Pentane (≥ 99%, anhydrous) and cyclopentane (> 98%, anhydrous) (used in a volume ratio of 50:50 *v*/*v*) supplied by Merck KGaA were used as a blowing agent. Triphenylsilane was purchased from abcr GmbH Company. Sodium hydroxide (pellets, anhydrous), methanol (≥ 99.9%, anhydrous), ethanol (≥ 99.9%, anhydrous), and maleic anhydride (99%) were provided by Sigma-Aldrich Corporation. Commercial bleached kraft softwood (eucalyptus) pulp was used in the experiments. Pulp was delivered in the form of dry sheets. The average moisture content was 4.3%. Initial pulp parameters are DP—1090, α-amylase content—85.8%, average fibres length—0.7 mm, primary fines content—10%.

### 2.2. Chemical Modifications of Eucalyptus Fibers

**Figure 1:**
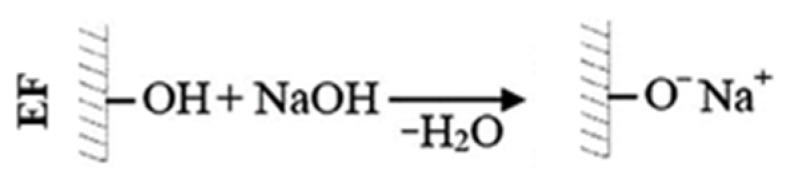
Alkali treatment of eucalyptus fibers.

Alkali treatment (Figure 1): Pre-dried eucalyptus fibers were soaked in 5% sodium hydroxide solution for 30 min. After alkali treatment, the immersed fibers were washed with distilled water. Finally, the eucalyptus fibers were neutralized with a 1% acetic acid solution. The alkali-treated fibers were vacuum-dried at 80 °C for 24 h.

**Figure 2:**
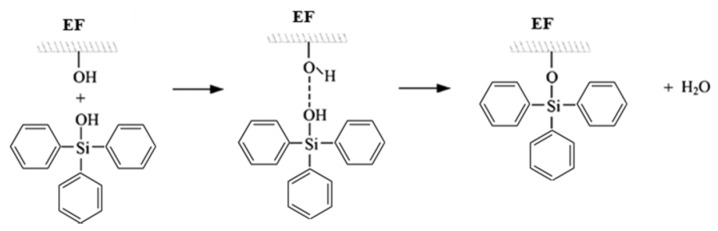
Silane treatment of eucalyptus fibers.

Silane treatment (Figure 2): Silane solution was prepared by dissolving triphenylsilanol in ethanol. Pre-dried eucalyptus fibers were added to 5% of such prepared silane solution and treated with ultrasounds for 3 h. After this, the silane solution was evaporated and silane-treated eucalyptus fibers were vacuum-dried at 80 °C for 24 h.

**Figure 3:**
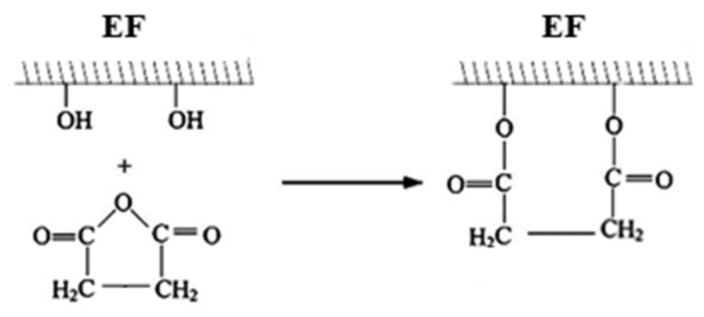
Maleic anhydride treatment of eucalyptus fibers.

Maleic treatment (Figure 3): Pre-dried eucalyptus fibers were reacted with 10 wt% of maleic anhydride at a temperature of 60 °C for 3 h. After maleic treatment, the eucalyptus fibers were washed with acetone solution to remove unreacted maleic anhydride. After that, the maleic-treated eucalyptus fibers were washed with water and vacuum-dried at 80 °C for 24 h. 

### 2.3. Preparation of Polyurethane Foams

Firstly, the eucalyptus bleached kraft pulp was fragmented by cutting for small (5 × 5 mm) squares then minced in a knife homogenizer (MPW-120) by 2 min. Polyurethane foams were synthesized by mixing the two-components system. Firstly, polyol, catalysts, surfactant, and blowing agents were mixed for 60 s at 4500 RPM (rounds per minute). The obtained component A was modified with 2 wt% of chemically-treated eucalyptus fibers. Then, the mixture was homogenized for 60 s at 4500 RPM. After that, a calculated amount of component B (isocyanate) was added to the mixture, and both components were mixed for 10–20 s at 4500 RPM. The obtained mixture was poured into an open box and the foams were allowed to grow freely in the vertical direction. All prepared foams were cured at room conditions for 24 h. After that, the samples were cut and tested according to the selected standards. The selected amounts of all formulas are presented in Table 1.

**Figure 4:**
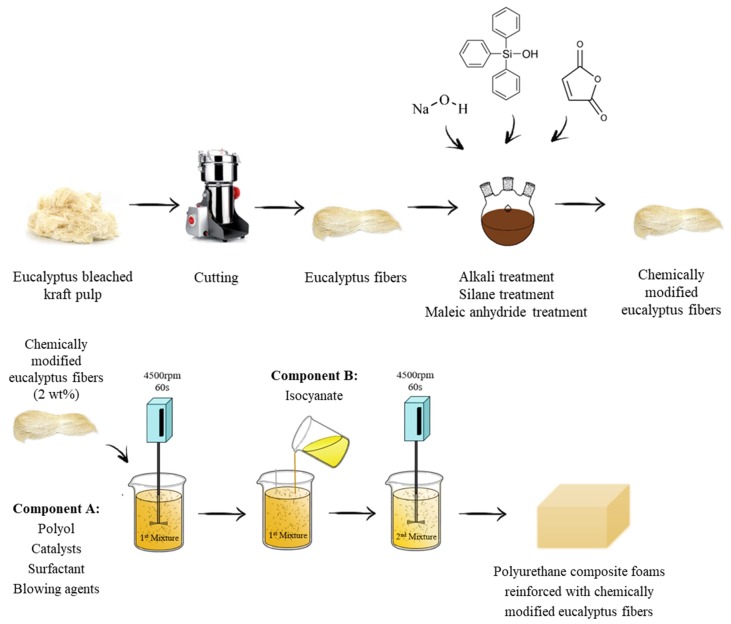
Schematic representation for the preparation of polyurethane foams modified with eucalyptus fibers.

Schematic representation for the preparation of polyurethane foams modified with eucalyptus fibers is presented in Figure 4.

### 2.4. Methods

The viscosity of the polyol systems was evaluated using a Viscometer DVII+ (Brookfield, Dresden, Germany) in the function of a shear rate according to ISO 2555. The measurement was performed in ambient temperature.

The chemical structure of eucalyptus fibers and polyurethane foams was evaluated using Fourier-transform Infrared Spectroscopy (FTIR). The measurements were performed using the Nicolet iS50 spectrometer (Thermo Fisher Scientific, Waltham, MA, USA) equipped in DGTS/KBr detector. 

The morphology of polyurethane foams was determined using scanning electron microscope JSM-5500 LV (JEOL Ltd., Tokio, Japan). The samples were scanned in the free-rise direction at the accelerating voltage of 10 kV. 

The apparent density of polyurethane foams was calculated as the ratio of foam mass to its volume according to ISO 845 standard.

The closed-cell content was determined according to PN-EN ISO 4590 using the helium pycnometer AccuPyc 1340 with the FoamPyc option (Micrometrics, Norcross, Norcross, GA, USA) in S.Z.T.K. ‘TAPS’ - Maciej Kowalski Company (Poland).

Compressive strength (*σ_10%_*) of polyurethane foams was evaluated according to ISO 844 standard. All samples were measured in the perpendicular and parallel direction to the foam growth direction using Zwick Z100 Testing Machine (Zwick/Roell Group, Ulm, Germany). The measurement was performed up to 10% of sample deformation (load cell of 2 kN, constant speed of 2 mm∙min^−1^).

Flexural strength (*ε_f_*) of polyurethane foams was evaluated according to ISO 178 standard. The measurement was performed using Zwick Z100 Testing Machine (Zwick/Roell Group, Ulm, Germany) at a constant speed of 2 mm∙min^−1^. 

Surface hydrophobicity of polyurethane foams was measured using contact angle goniometer OEC-15EC (DataPhysics Instruments GmbH, Germany) with software module SCA 20. The average of 10 measurements was evaluated. 

The thermal properties of polyurethane foams were determined by thermogravimetric analysis (TGA) using STA 449 F1 Jupiter Analyzer (Netzsch Group, City, Germany). The measurement was performed for samples of 10 mg. Samples were heated in an argon atmosphere up to 600 °C. The decomposition temperatures—T_2%,_ T_10%,_ T_50%,_ and T_80%_ were determined. The results of thermogravimetric measurement were presented as a percentage weight loss as a function of temperature—the TGA curve and first derivative of the TGA curve (DTG) for determining the inflection points.

Water absorption of polyurethane foams was performed according to ISO 2896. Samples were weighed (*m*_0_) and immersed in distilled water for 24 h (water depth of 1 cm). After this, polyurethane foams were weighed again (*m*) and the water absorption was calculated following Equation (1).

*WA* = *m* − *m*_0_/*m*_0_ (1)

The thermal conductivity of polyurethane foams was measured using the heat flow meter apparatus LaserComp 50 (TA Instruments, New Castle, DE, USA) with a 2.5 cm × 2.5 cm size heat flow transducer. The upper and lower plates of the HFMA instrument were set with a mean temperature of 25 °C. 

The fire behavior of polyurethane foams was analyzed using a cone calorimeter apparatus according to ISO 5660 in S.Z.T.K. ‘TAPS’—Maciej Kowalski Company (Lodz, Poland). Each specimen with dimensions of 100 × 100 × 25 mm^3^ was wrapped with aluminum foil and burned at an external heat flux of 35 kW m^−2^. The parameters were recorded during the time. 

## 3. Results and Discussion

### 3.1. Characterization of Eucalyptus Fibers

**Figure 5:**
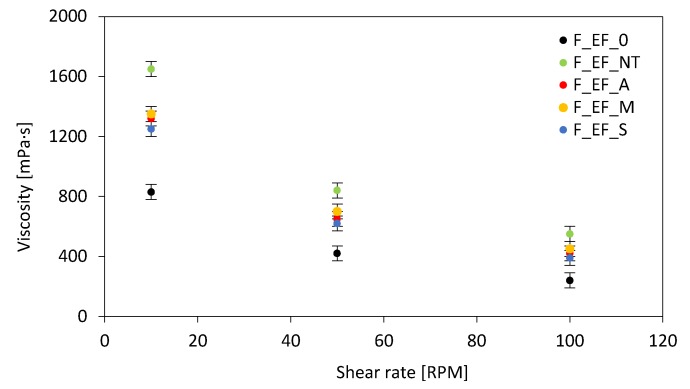
The viscosity of the polyol system modified with eucalyptus fibers in the function of the shear rate.

The results of the dynamic viscosity of polyol mixtures with the addition of 2 wt% of eucalyptus fibers are presented in Figure 5. In all cases, the dynamic viscosity increases significantly with the addition of the fibers. The highest value of dynamic viscosity exhibits polyol systems modified with non-treated eucalyptus fibers, which is almost twice as high as that of polyol systems containing chemically-treated fibers. Thus, the effect of the fibers on the pseudo-plasticity behavior becomes more significant for mixtures with the addition of non-treated fibers, leading to highly non-Newtonian behavior. The difference between the dynamic viscosity of the modified systems indicates that the chemical modification of eucalyptus fibers disturbs the formation of larger agglomerates and distributes fibers more evenly in polyol mixture, which results in lower viscosity of the modified systems. In all cases, the dynamic viscosity decreases in the function of the shear rate. The dynamic viscosity of all modified systems decreases dramatically at the beginning of the shearing. Once the filler molecules reach the best possible arrangement, the value of viscosity becomes relatively stable, which is typical for non-Newtonian fluids and is well described in previous studies [61,62]. Similar results were reported by Kairyte et al. [63] in the case of PUR foams modified with titanate-coupled paper waste sludge particles. It was determined that the application of a titanate-coupled agent reduces the dynamic viscosity of PUR modified mixtures by approximately 2.3 times when compared with PUR mixture containing non-coupled particles. The reduced viscosity was assigned as the operating principle of the titanate-coupled agent, which was based on the unripping of agglomerates and even distribution of particles in PUR mixtures. Similar results were found in the case of silane-coupled ZnO [64] and talc [65] particles.

**Figure 6:**
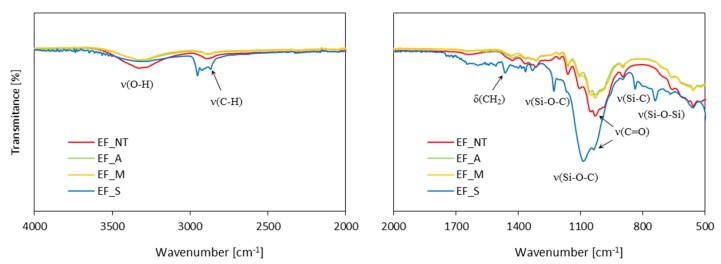
Fourier-transform Infrared Spectroscopy (FTIR) spectra of chemically-treated eucalyptus fibers.

The FTIR spectra of eucalyptus fibers, before and after the chemical modifications, are presented in Figure 6. An intense band localized at 3310 cm^−1^ corresponds to O-H stretching vibration [66]. The band observed at 2880 cm^−1^ is characteristic for C-H stretching vibration. The low intense band localized at 1027 cm^−1^ is attributed to carbonyl stretching vibration (C=O) and indicates the presence of hemicellulose in the eucalyptus fibers [67]. The band appearing at 1639 cm^−1^ is characteristic of the absorbed water. Bands localized at 1425, 1322, and 1034 cm^−1^ indicate the presence of lignin and correspond to CH_2_ bending vibration, O-H bending vibration and C-O stretching vibration of acetyl group, respectively. β-glycosidic bonds between carbohydrate molecules in cellulose and hemicellulose is confirmed by the presence of a band at 892 cm^−1^ [68]. 

In general, the FTIR spectra of alkali-treated and maleic anhydride-treated eucalyptus fibers presented are very similar to that of the non-treated fibers. There is a reduced intensity of the band at 3310 cm^−1^ which corresponds to OH stretching vibration confirms the maleation of eucalyptus fibers [38,69]. The FTIR spectra of silane-treated eucalyptus fibers confirm the silanization of the fibers. The characteristic bands localized at 740 and 835 cm^−1^ correspond to Si-O-Si stretching vibration and Si-C stretching vibration, respectively [70,71]. Bands localized at 1080 and 1227 cm^−1^ are characteristic for Si-O-C vibration and confirm the successful reaction between silane and cellulose of eucalyptus fibers [70]. 

**Figure 7:**
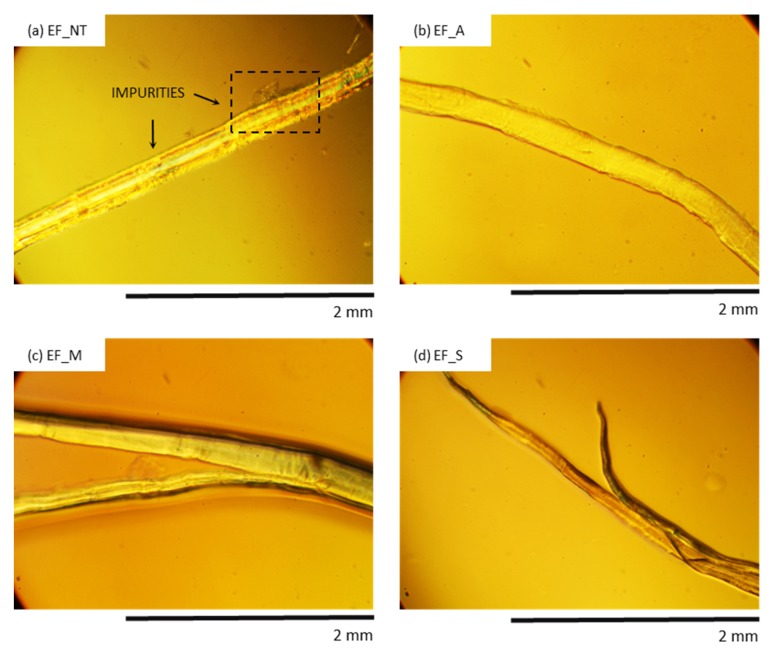
Optical image of (**a**) EF_NT, (**b**) EF_A, (**c**) EF_M and (**d**) EF_S observed at a magnification of 200.

The surface morphology of eucalyptus fibers before and after chemical treatments is shown in Figure 7. Non-treated eucalyptus fibers exhibit many impurities on the surface. After the alkali treatment, the surface of the fibers becomes clean and smooth. It indicates that the alkali treatment removes all impurities from the surface of the fibers. A similar effect is observed in the case of silanized and maleated fibers. It can be observed that chemical treatments improve the quality of the surface and all external impurities are removed. This observation is in agreement with previous works [72,73].

**Figure 8:**
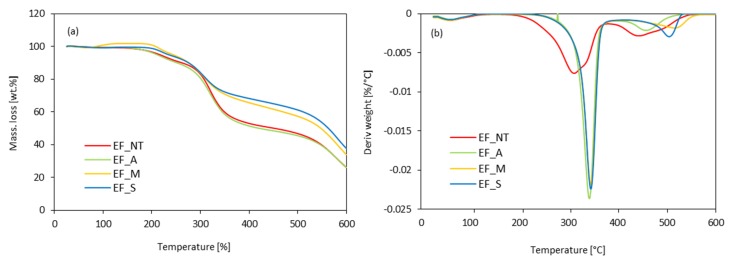
(**a**) thermogravimetric analysis curve (TGA) and (**b**) first derivative of the TGA curve (DTG) obtained for polyurethane foams modified with eucalyptus fibers.

The thermal degradation of eucalyptus fibers was evaluated employing TGA analysis (Figure 8). It can be observed that the eucalyptus fibers present three degradations steps. In the first step, between 50 and 130 °C, the moisture in the sample was evaporated, resulting in weight loss [74]. A second degradation step occurs around 200 °C and is attributed to the decomposition of cellulose, hemicellulose, and lignin [75,76]. After the main pyrolytic process, there is a slow and continuous degradation between 400 °C and 600 °C, attributed to lignin degradation (step III). The chemical modification significantly improved the thermal stability of eucalyptus fibers. The thermal degradation of non-treated fibers occurs at 307 °C, while for chemically modified fibers, the thermal degradation is extended to ~345 °C. Such improvement may be attributed to the partial removal of lignin and hemicellulose from the fibers, which are characterized by a lower thermal stability than cellulose [74]. In addition, the residue of non-treated carbon at ~600 °C is about 26%, while the residue modified with maleic anhydride or silane is significantly increased. For example, silanization treatment increases the value of the carbonized residue by up to 37%. Only alkali treatment did not improve the thermal stability of eucalyptus fibers.

### 3.2. Foaming Kinetic of Polyurethane Foams

The foaming process was determined by measuring the characteristic processing times, like start, growth, and tack-free time. The start time was measured from the start of the mixing of components to the start of a visible foam growth, growth time elapsing until we reached the highest volume of the foam, and the tack-free time was determined as the time when the foam solidifies completely and the surface is no longer tacky. 

**Figure 9:**
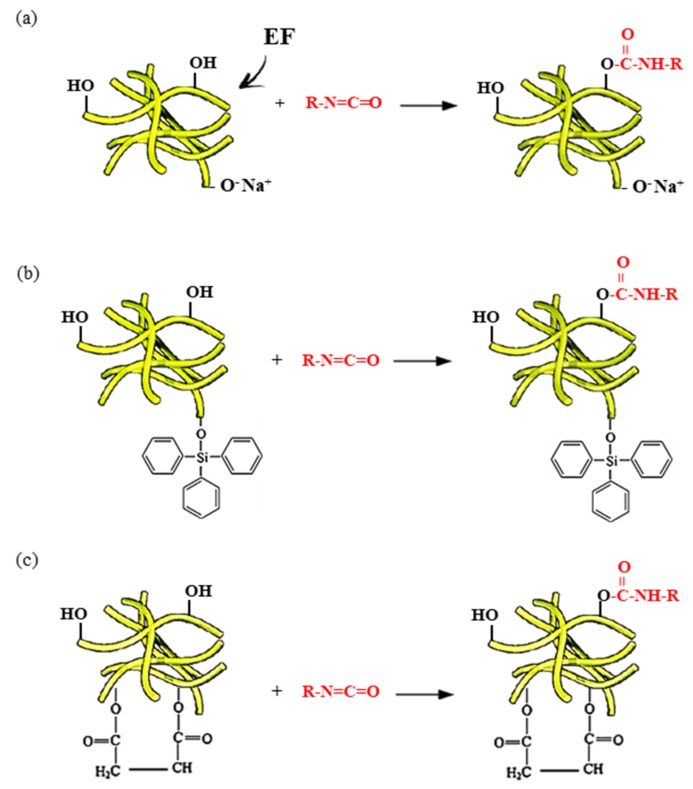
The possible mechanism between (**a**) alkali-treated, (**b**) silane-treated, (**c**) maleic-treated eucalyptus fibers and isocyanate groups.

The results presented in Table 2 indicate that the addition of eucalyptus fibers affects the foaming kinetics of polyurethane mixtures. Compared to unfilled F_EF_0, the addition of eucalyptus fibers increases the start and growth times. Previous studies have stated that, during the nucleation process, the addition of organic and inorganic fillers may act as additional nucleating centers. This results in the formation of a greater number of air bubbles. In addition, fiber surface treatment changes the surface nature as a result of removing some lignin and hemicellulose from the surface, and completely removing pectin, wax, oils, and other organic compounds. After removal, it is thought that the fibers have more cellulose particles exposed on the surface, which improves the adhesion of the fibers to the polymer matrix due to the greater number of possible reaction sites. Surface roughness also increases, which improves mechanical properties and adhesion [77]. Moreover, chemical treatment introduces new functional groups (Figure 9). The additional groups may react with reactive -NCO groups and affect the proper stoichiometry of polyurethane synthesis [14,78,79,80]. Because of this, the number of -NCO groups remaining to react with water is lower and the amount of produced CO_2_ is reduced. The expansion of formed cells is disturbed, leading to prolonged start and growth times. On the other hand, the further expansion of formed cells is additionally limited by an increased viscosity of modified PUR systems. The mobility of the molecules is reduced, which affects the polymerization kinetic of PUR synthesis. The rate of isocyanate conversion is decreased and the rate of polymerization of the polyurethane is slowed down. This results in prolonged start and growth times [81,82]. Comparing polyurethane systems containing fibers, the highest values of processing times are observed for the mixture modified with non-treated fibers. This indicates that the chemical modifications of eucalyptus fibers affect the chemical interaction between the surface of chemically-treated fibers and isocyanate during the foaming step. Similar results were also found in previous work [83,84]. 

### 3.3. Cellular Structure of Polyurethane Foams

**Figure 10:**
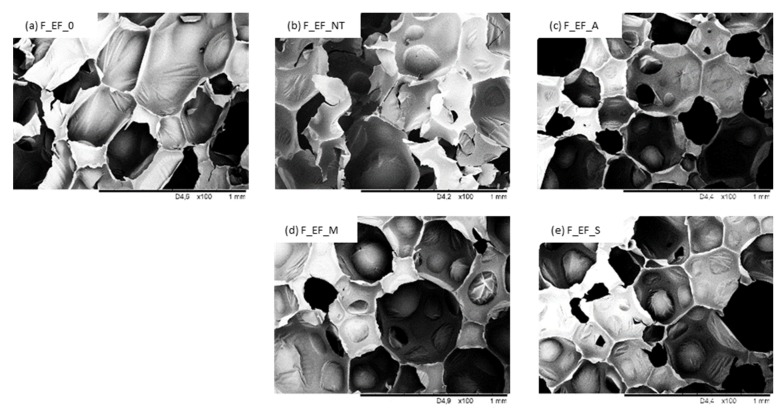
The morphology of (**a**) F_EF_0, (**b**) F_EF_NT, (**c**) F_EF_A, (**d**) F_EF_M, (**e**) F_EF_S observed at a magnification of ×100.

**Figure 11:**
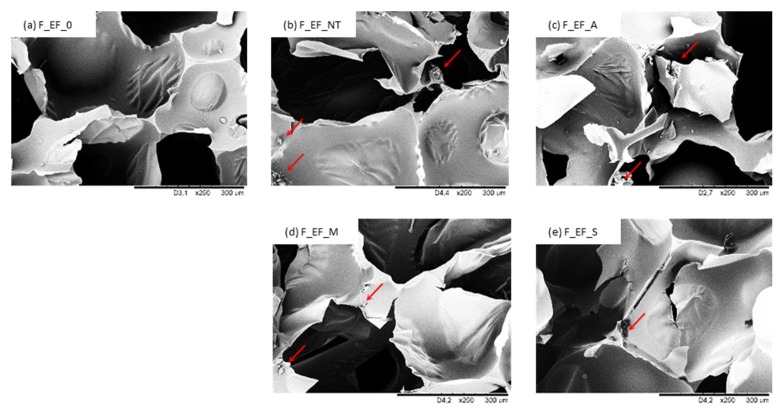
The morphology of (**a**) F_EF_0, (**b**) F_EF_NT, (**c**) F_EF_A, (**d**) F_EF_M, (**e**) F_EF_S observed at a magnification of ×200.

The cellular structure of polyurethane foams modified with eucalyptus fibers is shown in Figure 10 and Figure 11. The structure of non-treated F_EF_0 is homogenous and a high content of closed-cells is observed. The morphology of foams modified with non-treated eucalyptus fibers (F_EF_NT) is more heterogeneous and more open cells are observed in the foam structure. The porosity of F_EF_NT decreases from 91.5% to 89.2%. Such deterioration of the foam structure can be found in poor interfacial adhesion between eucalyptus fibers and the polymer matrix, which in turn disrupts the foaming process and creates a more defective structure [85]. As presented in Figure 11, eucalyptus fibers are not completely built in the structure, but some fibers are located in empty cells. This confirms the poor interfacial adhesion between fibers and the polymer matrix, which results in cell collapse and the formation of open pores in the structure [21]. The more uniform structure is observed in the case of foams with the addition of chemically-treated eucalyptus fibers. The overall structure of foams seems to be similar to the structure of F_EF_0. It can be concluded that the chemical modification of eucalyptus fibers can enhance the formation of more homogenous cells. The addition of chemically-treated fibers does not affect the porosity of the foam—a high value of porosity (>90%) is observed for all series of modified foams. This indicates that the chemical modification may improve the interphase adhesion between eucalyptus fibers and polymer matrix which results in the more effective development of polyurethane structure. The higher cross-linking degree of foams with the addition of chemically-treated fibers prevents disruption of the cells during the foaming process. Moreover, the incorporation of compatible fibers can form new edges which can encapsulate blowing agents as well as volatile substances released from the filler, thereby increasing the content of closed cells. 

The incorporation of eucalyptus fibers reduces the cell size of PUR foams, which is more prominent in the case of foams modified with chemically modified fibers (Table 3). This may be connected with the fact that after the chemical modification, the fibers have a more hydrophobic character which promotes a more intense nucleation process and reduces the average size of the cells [5,86,87]. In the case of foams modified with non-treated fibers, most of the pores are in the range of 480–500 µm, while the incorporation of modified fibers leads to foams with a more uniform distribution of cell diameters with an average value in the range of 420–445 µm.

Compared to F_EF_0, the apparent density increases with the addition of non-treated and chemically-treated eucalyptus fibers (Table 3). For example, the apparent density increases by ~10% for F_EF_NT, and by ~3% for F_EF_S. An increased apparent density of modified foams may be connected with greater viscosity of the polyurethane systems and limited expansion of bubble cells. The apparent density is additionally enhanced by the density of the fibers (the density of eucalyptus fibers is ca. 1.5 g∙cm^−3^). 

### 3.4. Mechanical Properties of Polyurethane Foams

The results of compressive strength (*σ_10%_*) are presented in Table 4. Compared to F_EF_0, the value of *σ_10%_* increases by ~5% for foams modified with non-treated eucalyptus fibers. The mechanical behavior increases after the chemical modifications. Alkali, maleic, and silane treatments of eucalyptus fibers resulted in an improvement of *σ_10%_* by ~11, ~15 and ~20%, respectively, compared to F_EF_0. The results reveal that mechanical behavior depends on the cellular structure of polyurethane foams. As shown in Figure 10, compared to foams modified with non-treated eucalyptus fibers, the incorporation of chemically-treated fibers results in the synthesis of foams with a more homogenous structure and a higher number of closed-cells, which provides superior support to withstand the compressive load. A reinforcing effect can be also provided by interfacial adhesion between modified fibers and polyurethane matrix, which facilitates the stress transfer. In the case of foams modified with non-treated fibers, the structure is less uniform and the mechanical properties of the obtained foams are deteriorated, even though the density increased due to the addition of the fibers. In addition, it should be pointed out that non-treated fibers exhibit a high tendency to agglomerate, which in turn leads to the interphase separation of the foam structure and promotes the failure of the cellular structure under the compressive load. Because of the many voids present in the structure of the polyurethane foams and non-uniform distribution of the fibers, the mechanical properties are slightly reduced, however, the obtained value *σ_10%_* is still larger than for F_EF_0. 

The incorporation of eucalyptus fibers influences flexural *σ_f_* and impact strength of foams as well (Table 4). Compared to F_EF_0, the abovementioned properties were improved for all series of foams containing modified eucalyptus fibers. Only the introduction of non-treated fibers into the foam resulted in a deterioration of these properties. The greatest improvement of the mechanical properties is observed for F_EF_S. Compared to F_EF_0, the *σ_f_* increases by ~6%, while the impact strength increases by ~48%. A slight decrease in mechanical behavior is observed for foams modified with non-treated fibers, F_EF_NT, which is associated with the agglomeration of eucalyptus fibers. According to the previous studies, the incorporated fibers can act as reinforcing centers and generate localized stresses under the action of a loading force. The aggregates of fibers may act as additional stress concentration centers that promote the cracking of the sample, leading to the deterioration of the mechanical properties [88,89]. The obtained results indicate that chemical modifications of eucalyptus fibers result in more effective stress transfer during the bending test. Additional groups present on the surface of the chemically-treated eucalyptus fibers increase compatibility between the fiber surface and the polyurethane matrix, which leads to the greater crosslinking of the foams. The elimination of voids and gaps in the cellular structure results in the better mechanical behavior of the foams modified with the addition of the chemically-treated fibers. 

### 3.5. Thermal Conductivity of Polyurethane Foams

The results presented in Table 3 indicate that compared to F_EF_0, the addition of non-treated eucalyptus fibers increases the value of thermal conductivity by ~8%. Contrary results are observed for foams with the addition of chemically-treated fibers. The thermal conductivity decreases by 8% for F_EU_M and F_EU_S. Such changes in thermal conductivity are mostly related to changes in closed-cells content and the size of cells. A possible explanation may be also found in different polarities of fibers and polymer, which results in poor compatibility and weak ability of the structure’s cells to encapsulate blowing gas [28,90]. This dependence is more prominent for foams modified with non-treated fibers. As mentioned earlier, non-treated fibers tend to agglomerate, leading to some destruction of the cell structure and a higher content of open cells. Due to this, the thermal conductivity of polyurethane foams is increased. The chemical treatment of fibers leads to better adhesion between fiber surfaces and the polyurethane matrix, which results in a more uniform structure. The well-dispersed fibers located in the cell struts prevent heat transfer by blocking radiation, acting as diffusion barriers. This, in turn, suppresses thermal radiation through processes of scattering or absorption, improving the insulating properties of resulting foams. After seven days, the thermal conductivity of foams modified with non-treated fibers increases to 0.036 W m^−1^ K^−1^, while for foams containing modified fibers, the values ranged from 0.030 to 0.034 W m^−1^ K^−1^. According to ASTM E170, all series of modified foams exhibit lower thermal conductivity than the maximum thermal conductivity required for commercial thermal insulation boards.

### 3.6. Contact Angle and Water Uptake of Polyurethane Foams

**Figure 12:**
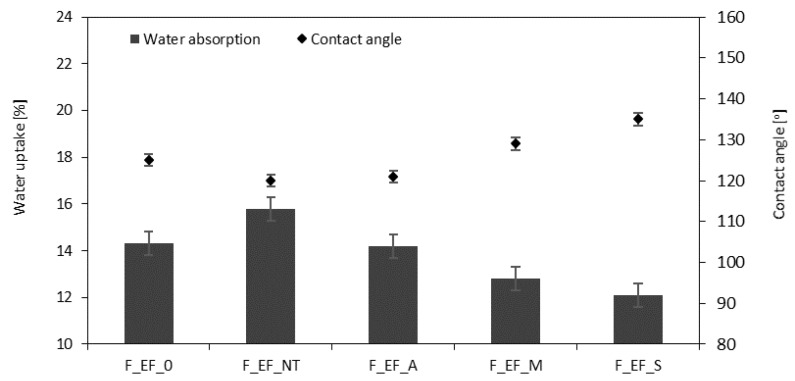
Water uptake of polyurethane (PUR) foams.

**Figure 13:**
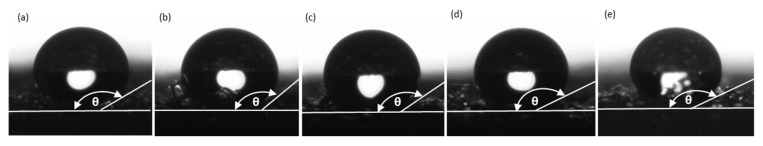
The contact angle of (**a**) F_EF_0, (**b**) F_EF_NT, (**c**) F_EF_A, (**d**) F_EF_M, (**e**) F_EF_S.

From an application point of view, the water uptake is an important property of polyurethane foams. Water uptake depends on the morphology of foams as well as the hydrophobic character of the filler. The results presented in Figure 12 indicate that the incorporation of non-treated eucalyptus fibers increases the water uptake. This may be connected with the more inhomogeneous open-cell structure of polyurethane foam. Based on SEM images (see Figure 10), the addition of non-treated eucalyptus fibers results in the opening of the foam cells. Moreover, fibers tend to agglomerate, which results in the creation of paths available to the water molecules to penetrate the foam structure [6,28,91,92]. The incorporation of fibers with a hydrophilic nature affects the water uptake also. Because of this, the character of foams modified with non-treated fibers becomes more hydrophilic, which is also confirmed by the reduced value of the contact angle (*θ*) (Figure 13). When compared with F_EF_0, the incorporation of non-treated eucalyptus fibers decreases the value of contact angle from 125° to 120°. Among the studied foams, the most hydrophobic character exhibit F_EF_S. The value of the contact angle is 135°, while the water uptake decreases to 12.1% after 24 h. 

### 3.7. Thermogravimetric Analysis of Polyurethane Foams

**Figure 14:**
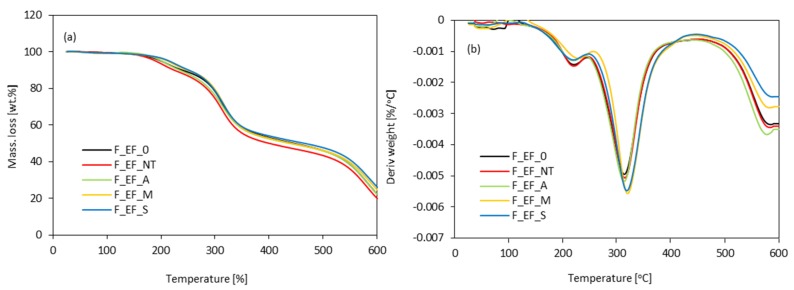
(**a**) TG and (**b**) DTG curves obtained for polyurethane foams modified with eucalyptus fibers.

The impact of the addition of eucalyptus fibers on polyurethane foams was evaluated by thermogravimetric analysis. Thermogravimetric (TG) profiles for all PUR foams are presented in Figure 14. The main findings are presented in Table 5.

The addition of non-treated and chemically-treated fibers results in a higher value of T_2%_. This indicates that the release of volatile products from the polyurethane matrix increases with the addition of the fibers [93]. Taking this into account, the volatile products of biodegradable fibers tend to release at lower temperatures, the higher values of T_2%_ may be connected with partial crosslinking between filler’s functional groups and isocyanate groups (-NCO) [94]. This effect is more prominent for foams modified with chemically-treated fibers. 

It is observed that PUR foams modified with chemically-treated fibers exhibit slower degradation in the temperature of ~200 °C, which corresponds to the decomposition of the urethane bond [95,96]. This indicates a higher crosslinking degree of foams modified with fibers. Compared to F_EF_0, the addition of chemically-treated fibers increases T_10%_ by 1–4 °C, depending on the modification. A contrary result is observed for F_EF_NT—the value of T_10%_ is reduced by ~3 °C. 

The enhancement of thermal stability of foams modified with eucalyptus fibers can be confirmed based on the results of T_50%_, which correspond to the structural decomposition of organic chains (mainly urea groups) [97,98,99]. Based on the results, it can be concluded that the addition of chemically-treated fibers improves the thermal stability of the obtained foams. Foams modified with non-treated fibers start to degrade at 430 °C, while the PUR foams modified with chemically-treated fibers start to degrade in the range of 459–468 °C. The amount of char residue for F_EF_0 is 24.4% at 600 °C. For F_EF_NT and F_EF_A the amount of char residue decreases to 22.3%. Contrary results are observed for F_EF_M and F_EF_S—the value of char residue increases to 26.6% and 28.3%, depending on the modification. The improvement in thermal stability may be associated with strong interfacial interaction between the PUR structure and fibers phases. Additionally, chemically-treated fibers can act as a cross-linker between PUR backbones, consequently enhancing the thermal properties of the foam. Tian et al. [94] reported that the enhancement of thermal stability of the soy-protein enhanced foams is may be attributed to the crosslinking effect of soy protein on the matrix, which prevents the volatile products generated during thermal decomposition, leading to the retardance of the thermal degradation action. A similar effect can be found in the presented work. 

### 3.8. Flammability of Polyurethane Foams

The cone calorimeter test provides a quantitative analysis of the flame retardancy of polyurethane foams. The results of ignition time (IT), peak heat release rate (pHRR), total heat release (THR), total smoke release (TSR), as well as an analysis of CO (COY) and CO_2_ (CO_2_Y) during the combustion and char residue are presented in Table 6. 

The IT of eucalyptus fibers incorporated PUR foams exhibit a slight increase in comparison with controlled F_EF_0. The highest value of IT is obtained for F_EF_S—the incorporation of silanized eucalyptus fibers increases IT from 5 to 7 s. 

**Figure 15:**
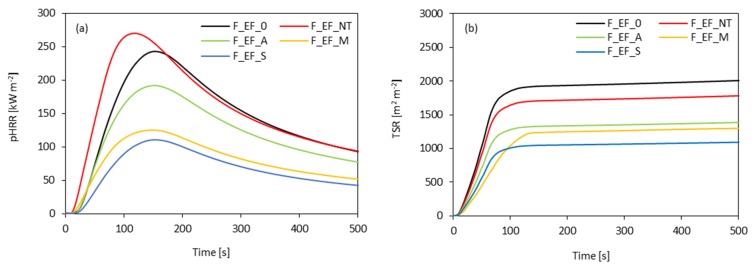
(**a**) peak heat release rate (pHRR) and (**b**) total smoke release (TSR) values obtained for polyurethane foams modified with eucalyptus fibers.

The intensity of the fire is correlated with the pHRR results. The results presented in Figure 15a, indicate that the incorporation of non-treated fibers has an impact on pHRR. When compared with F_EF_0, the value of pHRR increases by approx. 10% for F_EF_NT. However, a two-fold better performance can be observed for foams containing chemically modified fibers, i.e., silanized eucalyptus fibers reduce the pHRR value by approx. 55%. The reduction of the pHRR value may be related to the formation of a stable char layer on the polymer surface and releasing non-combustible gas. It should be pointed out that in the case of PUR foams used as an insulating material, the accessible value of pHRR is 300 kW m^−2^ [100], while for polyurethane foams modified with chemically-treated fibers, the value of pHRR does not exceed 190 kW m^−2^. 

The discussed results are in agreement with the total smoke release (TSR) results (Figure 15b). The addition of non-treated and chemically-treated fibers affects the value of the tested parameter. Non-treated fibers decrease TSR value from 1900 m^2^ m^−2^ to 1850 m^2^ m^−2^. Similar behavior is observed in the case of chemically-treated fibers—the value of TSR decreases by ca. 32%, 35%, and 45% for F_EF_A, F_EF_M, and F_EF_S, respectively. Table 6 shows that THR also decreased upon the incorporation of chemically-treated fibers. The lowest value of THR is ascribed to polyurethane foams modified with silanized fibers, which is reduced by ca. 9% as compared to F_EF_0. Moreover, as presented in Table 6 compared with F_EF_0, the addition of chemically-treated fibers decreases the COY and CO_2_Y values for all modified foams. This result illustrates that chemically-treated fibers hinder the heat transfer and flame spread, therefore decreasing the intensity of the fire. This may be connected with the fact that the non-treated fibers are easily ignitable and light more PUR foams in the bottom. The chemically-treated fibers are harder to be ignited and the char residue can suppress the smoke.

The results of the limiting oxygen index (LOI) are illustrated in Table 6. The addition of non-treated eucalyptus fibers decreases the LOI. Compared to F_EF_0, the value of LOI decreases from 20.2% to 19.8%. Contrary results are observed for PUR foams modified with chemically-treated fibers. In each case, the addition of modified fibers improves the LOI of the obtained foams. The greatest improvement in the value of LOI is obtained for F_EF_S-the LOI value increases to 22.1%. The overall effect proves that the silane treatment of fibers with triphenylsilanol enhances the fire resistance of PUR foams, because of the presence of phenyl groups with good thermal stability. Similar results were reported by other authors. For example, Cheng et al. [101] synthesized polyurethane foams enhanced with treated by a flame retardant ramie fibers. The authors have shown that the addition of 0.2 wt%–0.8 wt% of modified ramie fibers results in the drop of LOI, due to the carbonization of the fibers and formation of pores, which prevents the flame propagation in the matrix. 

## 4. Conclusions

This study aims to evaluate the influence of maleic anhydride, alkali, and silane (triphenylsilanol) treatments on the eucalyptus fibers and their polyurethane foam composites. The Fourier transform infrared spectroscopy (FTIR), optical microscopy, and thermogravimetric analysis (TGA) tests were carried out to understand the influence of the treatments on the fibers. FTIR results revealed that the chemical modifications of eucalyptus fibers were successfully performed. The results obtained in this study confirm that the addition of non-treated fibers in an amount of 2 wt% led to samples with worsened thermal stability and deteriorated insulating properties. Moreover, the samples were characterized by better flammability and higher water uptake as compared with unfilled foams. The incorporation of 2 wt% of non-treated fibers resulted in an improvement of compressive strength by ~5%, however, the flexural and impact strength decreased by 9% and 26%, respectively. Mechanical properties, thermal stability, and flammability tests showed that the obtained polyurethane foams with alkali-treated fibers are characterized by almost the same properties as unfilled foams. Contrary results were received for polyurethane foams containing maleic- and silane-treated fibers. The compressive, flexural, and impact test results confirmed that the silane-treated fiber composites displayed the highest properties than the other treated fiber composites. For example, the incorporation of 2 wt% of silanized fibers resulted in improvement of mechanical, flexural, and impact strength by ~20%, ~7%, and ~49%, respectively. Moreover, the thermal stabilities values showed the lowest decline for the silane-treated composites, due to the better thermal stability of the silane-treated fibers, and the lowest water absorption was also recorded for the silane-treated fiber composites. Furthermore, the flame resistance of the silane-treated fiber composites was also the best among the studied composites. The morphological studies confirmed that the silane-treated fiber composite had a stronger fiber/matrix adhesion interface. This study shows that the incorporation of chemically-treated eucalyptus fibers improves the mechanical, thermal, and flame retardant properties of polyurethane foams, therefore eucalyptus fibers may be utilized as a unique filler with good mechanical and thermal properties. These eucalyptus fiber-incorporated polyurethane foams may be used for many applications in buildings.

## Figures and Tables

**Table 1:** Polyurethane foams formulations.

Component Name	F_EF_0	F_EF_NT	F_EF_A	F_EF_M	F_EF_S
	Parts by Weight (wt%)				
STEPANPOL PS-2352	100	100	100	100	100
PUROCYN B	160	160	160	160	160
Kosmos 75	6	6	6	6	6
Kosmos 33	0.8	0.8	0.8	0.8	0.8
Tegostab B8513	2.5	2.5	2.5	2.5	2.5
Water	0.5	0.5	0.5	0.5	0.5
Pentane/cyclopentane	11	11	11	11	11
Non-treated EF	-	2	-	-	-
Alkali-treated EF	-	-	2	-	-
Maleic anhydride-treated EF	-	-	-	2	-
Silane-treated EF	-	-	-	-	2

**Table 2:** Characteristic processing times of polyurethane foams modified with eucalyptus fibers.

Sample	Start Time [s]	Growth Time [s]	Tack-Free Time [s]
F_EF_0	54 ± 3	460 ± 8	350 ± 10
F_EF_NT	68 ± 1	540 ± 10	490 ± 12
F_EF_A	58 ± 2	510 ± 10	410 ± 10
F_EF_M	62 ± 3	515 ± 8	425 ± 11
F_EF_S	60 ± 2	505 ± 10	400 ± 8

**Table 3:** Structural parameters and thermal conductivity results of polyurethane foams modified with eucalyptus fibers.

Sample	Cell Size [µm]	Closed-Cell Content [%]	Apparent Density [kg m^−3^]	Thermal Conductivity (After 1 d) [W m^−1^ K^−1^]	Thermal Conductivity (After 7 d) [W m^−1^ K^−1^]
F_EF_0	480 ± 9	91.5	38 ± 1	0.025	0.034
F_EF_NT	420 ± 8	89.2	42 ± 2	0.027	0.036
F_EF_A	445 ± 9	92.5	40 ± 2	0.025	0.033
F_EF_M	440 ± 8	92.1	41 ± 2	0.023	0.034
F_EF_S	435 ± 8	93.1	39 ± 1	0.023	0.030

**Table 4:** The results of the mechanical properties of polyurethane foams modified with eucalyptus fibers.

Sample	Compressive Strength (Parallel) *σ_10%_* [kPa]	Compressive Strength (Perpendicular) *σ_10%_* [kPa]	Flexural Strength *σ_f_* [MPa]	Impact Strength [kJ m^−2^]
F_EF_0	260 ± 8	145 ± 9	0.405 ± 0.007	0.350 ± 0.004
F_EF_NT	273 ± 9	155 ± 9	0.370 ± 0.006	0.260 ± 0.004
F_EF_A	290 ± 9	160 ± 7	0.430 ± 0.004	0.400 ± 0.006
F_EF_M	298 ± 7	170 ± 8	0.420 ± 0.006	0.490 ± 0.007
F_EF_S	312 ± 8	170 ± 8	0.432 ± 0.006	0.520 ± 0.006

**Table 5:** The results of thermogravimetric (TG) and DTG analysis.

Sample	T_2%_ [°C]	T_10%_ [°C]	T_50%_ [°C]	T_80%_ [°C]	Char Residue (at 600 °C)	DTG [°C]	DTG [%/min]
F_EF_0	120	209	457	585	24.4	308	0.0050
F_EF_NT	122	206	430	581	22.3	309	0.0051
F_EF_A	131	210	459	586	22.3	311	0.0052
F_EF_M	135	213	460	587	26.6	320	0.0056
F_EF_S	140	217	468	588	28.3	322	0.0056

**Table 6:** Characteristic parameters describing the flammability of polyurethane foams.

Sample	IT [s]	pHRR [kW m^−2^]	THR [MJ m^−2^]	TSR [m^2^ m^−2^]	COY [kg kg^−1^]	CO_2_Y [kg kg^−1^]	COY/ CO_2_Y	LOI [%]
F_EF_0	4	245	21.0	1900	0.20	4.05	0.050	20.2
F_EF_NT	3	270	22.5	1850	0.23	4.45	0.052	19.8
F_EF_A	6	190	20.2	1300	0.16	1.70	0.094	20.8
F_EF_M	7	120	19.4	1240	0.11	1.20	0.091	21.6
F_EF_S	7	110	19.3	1040	0.12	1.18	0.100	22.1
